# Glycosylation gene-based molecular recognition model for diabetic retinopathy

**DOI:** 10.3389/fmed.2026.1721094

**Published:** 2026-05-18

**Authors:** Qiong Wu, Pan Pan Ma

**Affiliations:** Department of Ophthalmology, Northwest Women’s and Children’s Hospital, Xi’an, Shaanxi, China

**Keywords:** biomarkers, diabetic retinopathy, glycosylation-related genes, molecular recognition model, verification

## Abstract

Diabetic retinopathy (DR) represents a significant public health challenge, with the potential to cause blindness and escalate healthcare costs, yet current diagnostic and therapeutic approaches remain insufficient. This study investigated the role of glycosylation-related differentially expressed genes (GRDEGs) in DR to identify novel biomarkers and therapeutic targets. Through analysis of Gene Expression Omnibus (GEO) datasets using differential expression analysis, functional enrichment, and machine learning, we identified 39 GRDEGs—including RPS23, VCAN, and ST8SIA4—that play significant roles in DR pathogenesis. These genes were enriched in biological processes such as wound healing and sphingolipid metabolism, as well as cancer-related pathways. Immune infiltration analysis revealed distinct patterns and correlations between immune cell types and GRDEGs, suggesting immune microenvironment involvement in DR progression. External validation using an independent blood dataset demonstrated moderate discriminatory performance (AUC 0.7–0.9), though this cross-tissue comparison should be interpreted as exploratory evidence of partial gene expression consistency rather than confirmation of biological mechanisms or clinical utility. Given the limited sample size and group imbalances in the discovery cohort, these results constitute proof-of-concept findings requiring validation in larger, balanced populations. Future research should focus on functional validation of identified GRDEGs and integration of the molecular recognition model into clinical workflows to enable proactive DR management.

## Introduction

Diabetic retinopathy (DR), a leading cause of blindness among working-age adults, results from chronic hyperglycemia-induced retinal microvascular damage, inflammation, and neurodegeneration (1). With the global prevalence of diabetes projected to reach 700 million by 2045 (2), early detection of DR is crucial. Although current fundus examination and imaging techniques (such as fundus photography) can effectively detect the early clinical signs of diabetic retinopathy (such as microaneurysms), these methods often fail to capture the early molecular or cellular changes that occur before the visible lesions appear. Therefore, the early molecular changes of the disease may be overlooked, leading to delayed diagnosis (3). Existing therapies—such as laser photocoagulation and anti-vascular endothelial growth factor (VEGF) agents (4)—cannot reverse established pathology, underscoring the urgent need for novel diagnostic and therapeutic strategies.

Glycosylation plays a crucial role in modulating DR-related processes, including inflammation, signaling, and apoptosis (5). Although dysregulation of glycosylation enzymes and glycan structures has been reported in the diabetic retina, the precise mechanisms by which altered glycosylation contributes to DR pathogenesis remain unclear (6). Systematic profiling of glycosylation-related differentially expressed genes (GRDEGs) is therefore crucial for identifying early biomarkers and potential therapeutic targets.

To address existing knowledge gaps, we integrated multicohort datasets from the Gene Expression Omnibus (GEO) to systematically screen for GRDEGs in DR. Advanced bioinformatics and machine learning pipelines were employed to develop a glycosylation-based molecular recognition model. This model demonstrated favorable discriminatory performance in the training dataset and retained moderate discriminatory ability in an independent blood dataset. Given the limited sample size of the discovery cohort and the tissue source discrepancy between the external validation set and training set, these findings should be interpreted as exploratory evidence suggesting that glycosylation-related gene signatures hold potential value for DR recognition, warranting further validation in larger, more balanced populations before clinical application.

## Materials and methods

### Data acquisition

The DR datasets GSE102485 (7, 8) and GSE185011 (9) were retrieved from the GEO database^1^ using the R package GEOquery (Version 2.70.0) (10, 11). Dataset GSE102485, derived from *Homo sapiens* retinal neovascularization membrane tissue, and dataset GSE185011, derived from *Homo sapiens* blood samples, were both downloaded as fragments per kilobase per million mapped reads (FPKM)-normalized RNA sequencing (RNA-Seq) expression data. Dataset GSE185011 was based on the GRCh38.p13 human reference genome (NCBI annotation release 109), and the corresponding annotation files were used in conjunction with this reference genome. The platforms for GSE102485 and GSE185011 were GPL18573 and GPL24676, respectively. The detailed characteristics of the dataset are provided in Table 1. GSE102485 contained 19 DR samples and 3 controls, whereas GSE185011 contained 5 DR samples and 5 controls. All DR and control samples from both datasets were included in this study.

**TABLE 1:** GEO Microarray chip information.

Attribute	GSE102485	GSE185011
Platform	GPL18573	GPL24676
Species	Homo sapiens	Homo sapiens
Tissue	Retinal neovascular proliferative membrane	Blood
Samples in DR group	19	5
Samples in Control group	3	5
Reference	PMID:31574534; PMID:35721704	PMID:35989592

Detailed information of the datasets GSE102485 and GSE18501. GEO, gene expression omnibus; DR, diabetic retinopathy.

Glycosylation-related genes (GRGs) were curated from the GeneCards database (12)^2^ using the keywords “Glycosylation” and “Protein Coding,” with a relevance score >5. These genes were subsequently validated using the PubMed database^3^ (13), resulting in a list of 732 non-redundant GRGs. Intersection with the expression data from GSE102485 and GSE185011 retained 684 unique GRGs (Supplementary Table 1).

The input data for this study were processed RNA-seq FPKM expression matrices from the GEO database. Following import into R software, expression value distributions were assessed to determine the necessity of log2 transformation. Subsequently, the normalizeBetweenArrays() function from the limma package (Version 3.58.1) (14) was applied to standardize expression distributions across samples. This step was performed to reduce overall distributional differences between samples and enhance comparability for subsequent differential analysis and model construction. The two datasets were preprocessed separately: GSE102485 was used for discovery set analysis and model construction, while GSE185011 was reserved for external validation. Boxplots were generated to evaluate overall changes in sample expression distributions before and after normalization.

All downstream analyses were conducted on the preprocessed FPKM expression matrices, with preprocessing focused on expression value transformation and inter-sample standardization rather than probe-level background correction. For cross-dataset analyses, batch effects were corrected using the ComBat method from the sva package.

### Glycosylation-related differentially expressed genes in diabetic retinopathy

Based on the grouping in GSE102485 (DR vs. control), differentially expressed genes (DEGs) were identified using the R package limma (Version 3.58.1) (8), with thresholds set at |logFC| >1 and adjusted *p*-value (adj.*p*) <0.05 (Benjamini–Hochberg [BH] correction). Genes with logFC >1 were classified as upregulated, while those with logFC <−1 were considered downregulated. The results were visualized using a volcano plot generated with the R package ggplot2 (Version 3.4.4). GRDEGs were obtained by intersecting DEGs with the curated GRG list and visualized using a Venn diagram. The top 20 GRDEGs were visualized in a heatmap (Version 1.0.12), and their chromosomal distributions were mapped using the RCircos package (15) (Version 1.2.2). After identifying GRDEGs, a diagnostic model was developed using a stepwise feature-selection strategy. First, univariate logistic regression was employed to assess associations between candidate genes and DR status, with *p*-values used for initial screening. Next, candidate genes were ranked using a random forest algorithm, and top-ranked features were selected based on MeanDecreaseGini values. LASSO regression was then applied for variable shrinkage and model construction, with the optimal penalty parameter λ determined through cross-validation. Final model genes were selected based on non-zero regression coefficients, and model risk scores were calculated by weighting the coefficients with gene expression levels. This sequential approach aimed to reduce overfitting while improving model stability and reproducibility.

### Gene ontology and kyoto encyclopedia of genes and genomes enrichment analysis

GO analysis (16) is a widely used method for comprehensive functional enrichment studies, encompassing biological processes (BPs), cell components (CCs), and molecular functions (MFs). The KEGG database (17) provides extensive information on genomes, biological pathways, diseases, and pharmaceutical compounds. In this study, GO and KEGG enrichment analyses of GRDEGs were conducted using the R package clusterProfiler (18) (Version 4.10.0). Enriched terms with adjusted *p* <0.05 (BH correction) and false discovery rate (*q*) <0.25 were retained for further analysis.

### Development of a molecular recognition model for diabetic retinopathy

Initially, GRDEGs were evaluated using logistic regression analysis to examine the relationship between the independent variables and the binary dependent variable (DR vs. control). A *p*-value threshold of ≤ 0.05 was used to identify GRDEGs that were significantly associated with DR. The collective expression of GRDEGs included in the logistic regression model was visualized using a forest plot.

Next, GRDEG expression was further analyzed. To develop the DR molecular recognition model based on the GSE102485 dataset, significantly differentially expressed GRDEGs were evaluated using the random forest (RF) algorithm. This ensemble learning method combines multiple decision trees through bootstrap aggregation (bagging). The model was built using the randomForest package (19), with parameters set to set.seed (2024) and ntree = 1000. The importance of each feature gene was assessed using MeanDecreaseGini values, which reflect the average decrease in impurity contributed by each variable across all decision trees. Higher MeanDecreaseGini values indicate greater relevance in classification. The top 10 genes were selected based on descending MeanDecreaseGini values for further filtering of GRDEGs.

Following initial feature screening using the random forest method, LASSO binary logistic regression analysis was performed with the glmnet package. The optimal penalty parameter was determined through cross-validation, yielding three final feature genes: CEP290 (coefficient: −7.083320), GNS (coefficient: 4.263735), and GRN (coefficient: 1.347877). The RiskScore is obtained by summing up the results of multiplying the expression levels of the final input genes with their corresponding regression coefficients. The risk score was calculated as:


RiskScore=(-7.08332×ExpCEP290)+

(4.263735×ExpGNS)+(1.347877×ExpGRN)

where ExpCEP290, ExpGNS, and ExpGRN represent the normalized expression levels of the respective genes.

### Validation of the molecular recognition model for diabetic retinopathy

The R package pROC (20) (Version 1.18.5) was initially used to generate the receiver operating characteristic (ROC) curve and to calculate the area under the curve (AUC) for the molecular recognition model, based on the GSE102485 dataset. This analysis evaluated the diagnostic efficacy of the model in detecting DR. AUC values ranging from 0.5 to 1.0 were interpreted as follows: 0.5–0.7 indicated low accuracy, 0.7–0.9 indicated moderate accuracy, and >0.9 indicated high accuracy. A nomogram (21) was constructed using the R package rms (Version 6.7–1) to visualize the relationships among the model genes. Calibration curves were used to assess the predictive accuracy of the LASSO-derived DR model. Additionally, decision curve analysis (DCA) plots, generated using the R package ggDCA (22) (Version 1.1), were employed to assess the clinical utility of the model in the GSE102485 dataset.

### Gene ontology functional similarity analysis

GO annotations (16) offer a measurable framework for evaluating functional similarities between genes and genomes, serving as a fundamental element for multiple bioinformatics analyses. In this study, functional similarity among the model genes was evaluated using the R package GOSemSim (23) (Version 2.28.0).

### Differential expression verification and ROC curve analysis

The expression levels of model genes in DR and control samples were compared using the training dataset (GSE102485) and validated in an independent dataset (GSE185011), with differences visualized using heat maps. ROC curves and corresponding AUC values were calculated using the pROC package (20) (Version 1.18.5). AUC values were interpreted as follows: >0.9 indicated high diagnostic accuracy, 0.7–0.9 indicated moderate accuracy, and 0.5–0.7 indicated low accuracy.

### Immune infiltration analysis

CIBERSORT (24), which employs linear support vector regression, was used to analyze transcriptome expression matrixes and estimate the types and proportions of immune cells in mixed cell populations. Using the LM22 signature, CIBERSORT deconvoluted the GSE102485 expression matrix, and samples with immune cell enrichment scores greater than zero were retained for analysis. The resulting immune cell infiltration matrix was visualized as a proportion bar chart. Correlations among immune cell types and associations between model genes and immune cell subsets were assessed using Spearman’s method and visualized using the R package pheatmap (Version 1.0.12) and ggplot2 (Version 3.4.4), respectively.

### Statistical analysis

All analyses were conducted using R software (Version 4.3.0). For comparisons between the two groups, continuous variables were analyzed using either the independent Student’s *t*-test for normally distributed data or the Mann–Whitney *U*/Wilcoxon rank-sum test for non-normally distributed data. Comparisons involving three or more groups were performed using the Kruskal–Wallis test. Correlations were assessed using Spearman’s rank correlation coefficient. A two-sided *p* <0.05 was considered statistically significant unless otherwise specified.

## Results

### Technology roadmap

**FIGURE 1:**
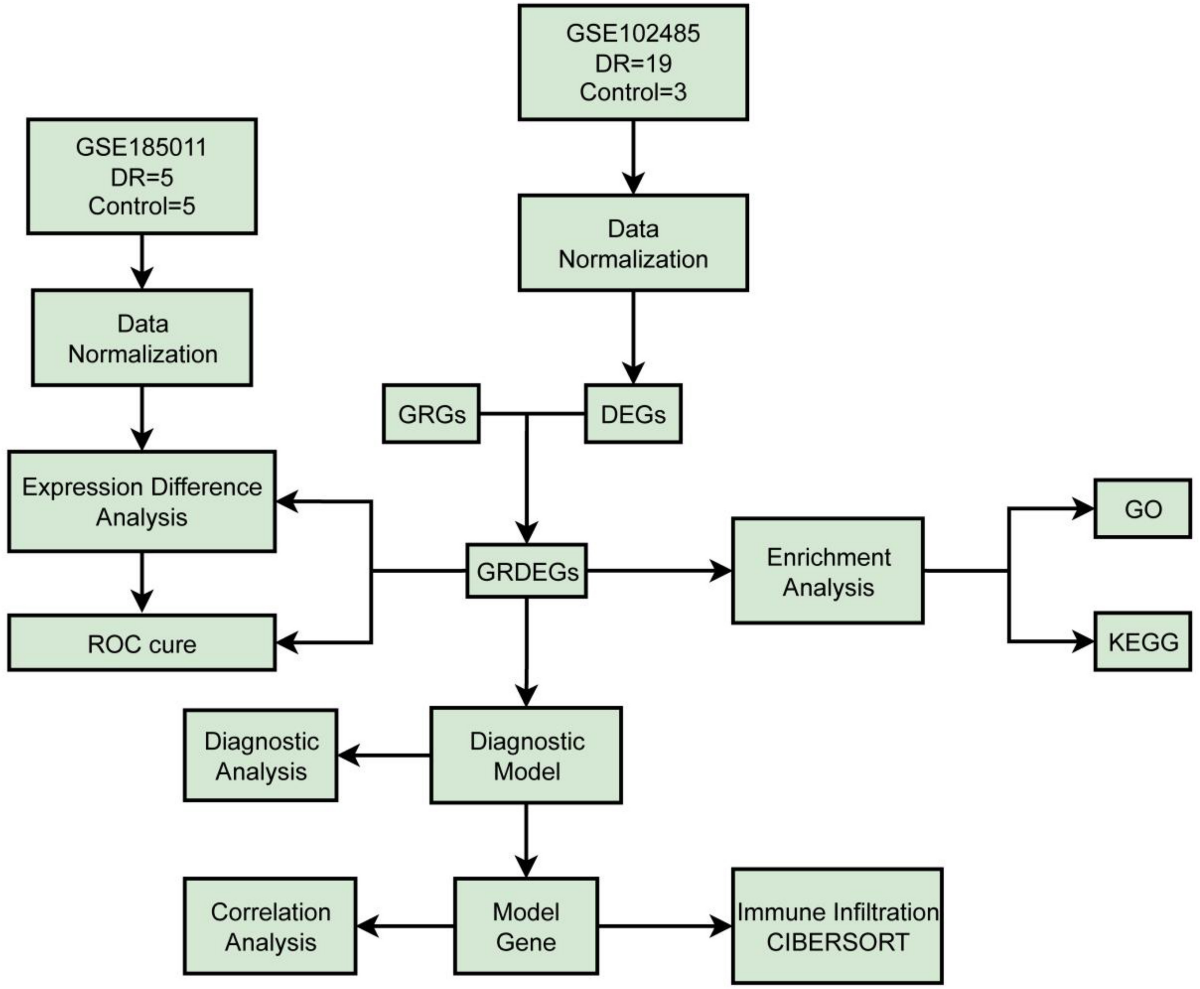
Technology roadmap. DR, diabetic retinopathy; DEGs, differentially expressed genes; GRGs, glycosylation-related genes; GO, gene ontology; KEGG, kyoto encyclopedia of genes and genomes; GRDEGs, glycosylation-related differentially expressed genes; ROC, receiver operating characteristic.

The technology roadmap is illustrated in Figure 1.

#### Merging of diabetic retinopathy datasets

**FIGURE 2:**
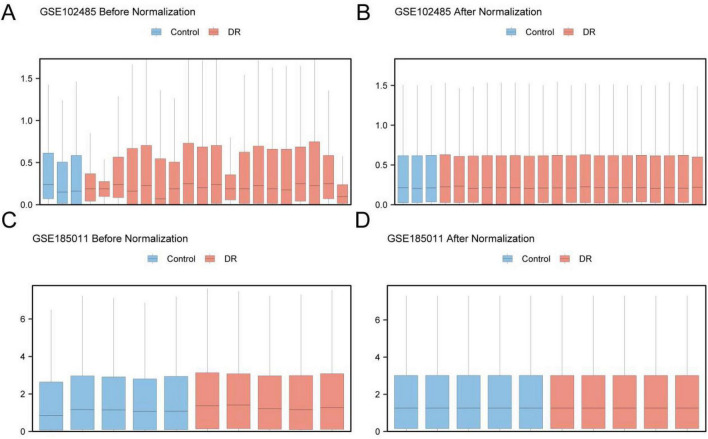
GSE102485 and GSE185011 normalization. **(A)** Boxplot of GSE102485 distribution in GEO dataset before normalization. **(B)** Boxplot of GSE102485 distribution of GEO dataset after standardized processing. **(C)** Boxplot of GSE185011 distribution of the dataset before normalization. **(D)** Boxplot of GSE185011 distribution of the data set after standardized processing. Orange is DR, blue is Control samples.

The R package sva (Version 3.56.0) was used to normalize the DR datasets GSE102485 and GSE185011. Following normalization, the finalized versions of the GEO datasets were retained under their original identifiers. Distribution boxplots (Figures 2A–D) were generated to evaluate expression values before and after normalization. The plots indicated that expression levels across samples became more consistent after normalization. The blood dataset (GSE185011) was used exclusively for independent external validation of the model and was not used as the primary data source for the main analyses.

#### Glycosylation-related differentially expressed genes in diabetic retinopathy

**FIGURE 3:**
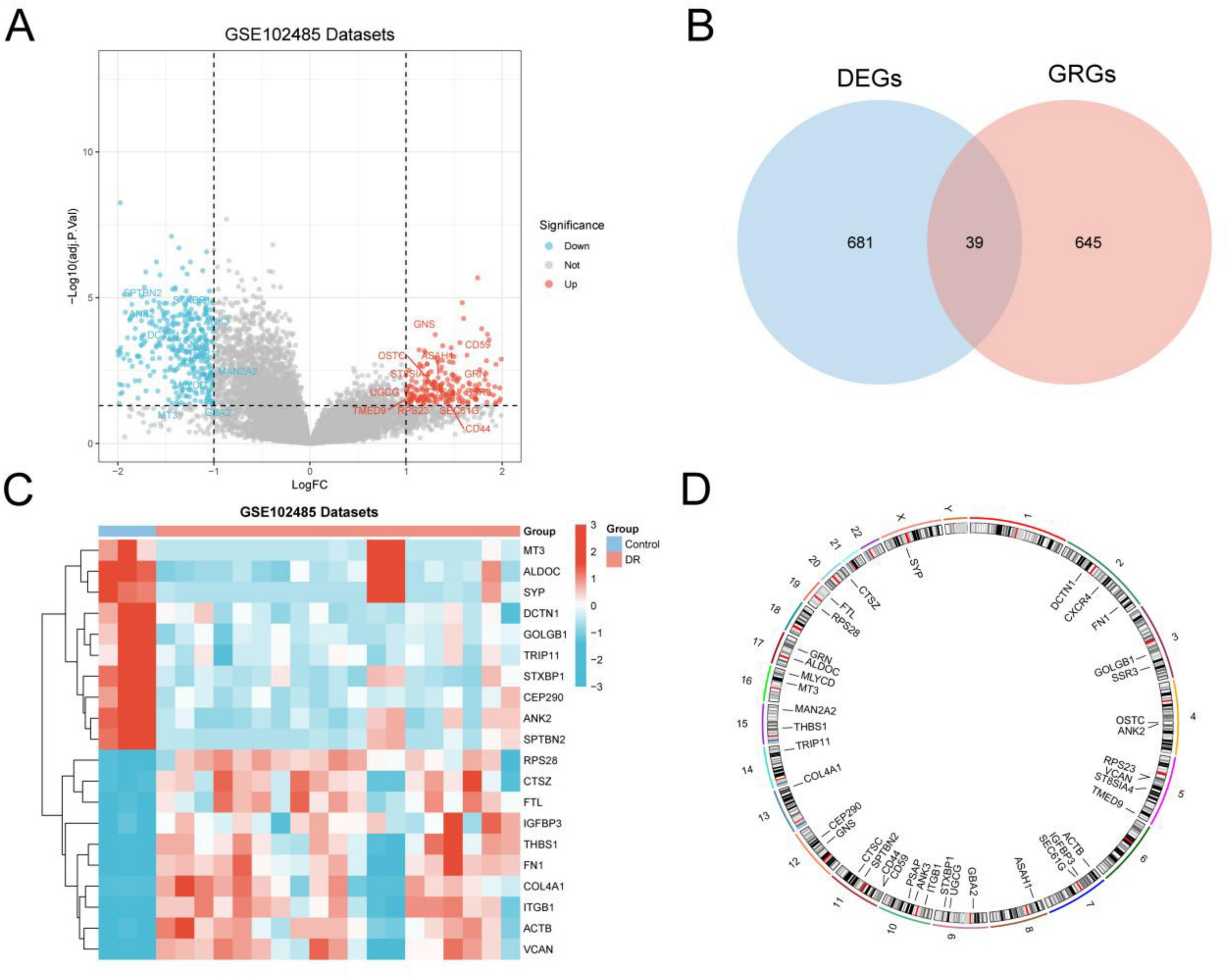
Differential gene expression analysis Volcano plot of differentially expressed genes analysis of DR group and Control samples in the **(A)** GEO dataset GSE102485. **(B)** Venn diagram of DEGs and GRGs in GEO dataset GSE102485. **(C)** Heat map of DEGs related to glycosylation in GSE102485 of GEO dataset. **(D)** Chromosomal mapping of GRDEGs. DR, diabetic retinopathy; DEGs, differentially expressed genes; GRGs, glycosylation-related genes; GRDEGs, glycosylation-related differentially expressed genes. Orange is DR and blue is Control samples. Red represents high expression and blue represents low expression in the heat map.

The GEO dataset GSE102485 was divided into two groups: DR and control. To examine the differential gene expression between the groups, the dataset was analyzed using the R package limma. A total of 720 DEGs were identified based on the thresholds of |logFC| >1 and an adjusted *p*-value (adj.*p*) <0.05. Among these, 257 genes were upregulated (logFC >1, adj.*p* <0.05), whereas 463 were downregulated (logFC <−1, adj.*p* <0.05). A volcano plot was generated to visualize these results (Figure 3A).

To identify GRDEGs, the DEGs (|logFC| >1, adj.*p* <0.05) were intersected with GRGs, and the overlap was illustrated using a Venn diagram (Figure 3B). This analysis yielded 39 GRDEGs (Supplementary Table 2). Subsequently, the expression patterns of GRDEGs across samples in the GSE102485 dataset were examined. The top 20 GRDEGs were visualized using a heat map generated with the R package pheatmap (Figure 3C). To explore chromosomal distribution, the R package RCircos was used to map the chromosomal locations of the 39 GRDEGs, resulting in a chromosomal localization plot (Figure 3D). Notably, a higher density of GRDEGs was observed on chromosomes 5 and 11, including *RPS23, VCAN*, *ST8SIA4*, *TMED9, CTSC*, *SPTBN2*, *CD44*, and *CD59.*

#### Gene ontology and kyoto encyclopedia of genes and genomes enrichment analysis

**FIGURE 4:**
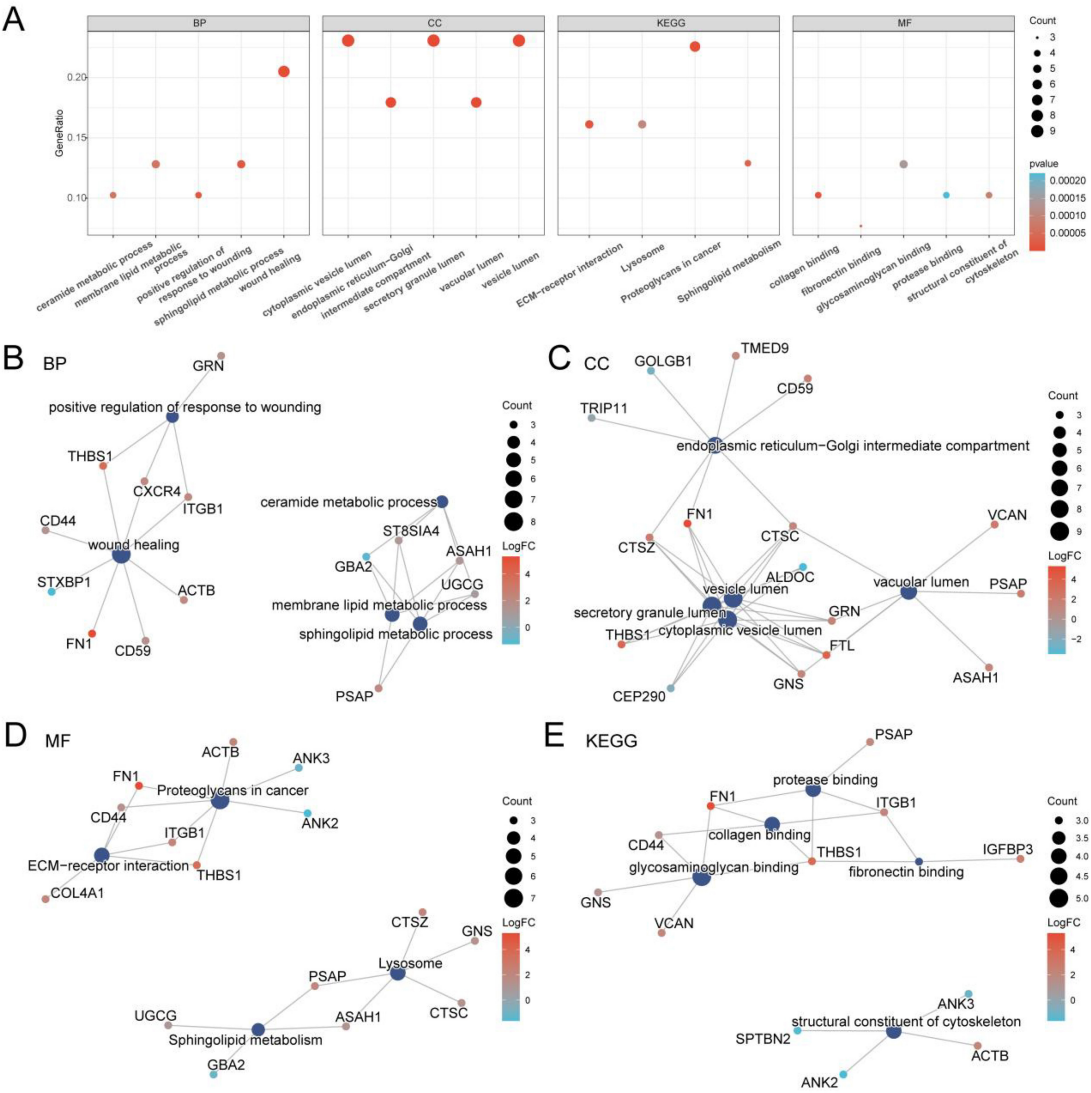
GO and KEGG enrichment analysis for GRDEGs. **(A)** Bubble plot of GO and KEGG enrichment analysis results of GRDEGs: BP, CC, MF and KEGG. GO terms and KEGG terms are shown on the abscissa. **(B–E)** GO and KEGG enrichment analysis results of GRDEGs network diagram showing: BP **(B)**, CC **(C)**, MF **(D)**, and KEGG **(E)**. The dark blue nodes represent entries, and the lines represent the relationship between entries and molecules. In the molecular nodes, red represents, and blue represents down-regulation. GRDEGs, glycosylation-related differentially expressed genes; GO, gene ontology; KEGG, kyoto encyclopedia of genes and genomes; BP, biological process; CC, cellular component; MF, molecular function. The screening criteria for gene ontology (GO) and pathway (KEGG) enrichment analysis were adj.*p* <0.05 and FDR value (*q*-value) <0.25.

GO and KEGG enrichment analyses were performed to explore the biological roles of the 39 GRDEGs in DR, focusing on BP, CC, MF, and associated KEGG pathways. The detailed results are presented in Table 2. The GO analysis revealed that the 39 GRDEGs were primarily associated with BPs such as wound healing and transplantation responses. They were also significantly enriched in metabolic processes, including sphingolipid metabolism, membrane lipid metabolism, and ceramide metabolism. In terms of CCs, the GRDEGs were primarily localized to the endoplasmic reticulum–Golgi intermediate compartment, secretory granule lumen, cytoplasmic vesicle lumen, vesicle lumen, and vacuolar lumen. For MFs, significant enrichment was observed in collagen binding, fibronectin binding, structural constituents of the cytoskeleton, glycosaminoglycan binding, and protease binding. Furthermore, KEGG pathway analysis revealed that the GRDEGs were enriched in pathways such as proteoglycans in cancer, extracellular matrix–receptor interaction, sphingolipid metabolism, and lysosomal pathways. The GO and KEGG enrichment results were visualized using a bubble diagram (Figure 4A).

**TABLE 2:** Results of GO and KEGG enrichment analysis for GRDEGs.

Ontology	ID	Description	GeneRatio	BgRatio	*p*-value	p.adjust	qvalue
BP	GO:0042060	Wound healing	8/39	423/18870	2.00E-06	2.97E-03	2.09E-03
BP	GO:1903036	Positive regulation of response to wounding	4/39	77/18870	1.89E-05	9.90E-03	6.95E-03
BP	GO:0006665	Sphingolipid metabolic process	5/39	162/18870	1.99E-05	9.90E-03	6.95E-03
BP	GO:0006643	Membrane lipid metabolic process	5/39	209/18870	6.73E-05	2.06E-02	1.44E-02
BP	GO:0006672	Ceramide metabolic process	4/39	107/18870	6.90E-05	2.06E-02	1.44E-02
CC	GO:0005793	Endoplasmic reticulum–Golgi intermediate compartment	7/39	134/19886	6.92E-09	5.82E-07	2.94E-07
CC	GO:0034774	Secretory granule lumen	9/39	322/19886	9.46E-09	5.82E-07	2.94E-07
CC	GO:0060205	Cytoplasmic vesicle lumen	9/39	325/19886	1.03E-08	5.82E-07	2.94E-07
CC	GO:0031983	Vesicle lumen	9/39	326/19886	1.05E-08	5.82E-07	2.94E-07
CC	GO:0005775	Vacuolar lumen	7/39	176/19886	4.57E-08	2.02E-06	1.02E-06
MF	GO:0005518	Collagen binding	4/39	68/18496	1.25E-05	1.91E-03	1.09E-03
MF	GO:0001968	Fibronectin binding	3/39	29/18496	3.05E-05	2.33E-03	1.33E-03
MF	GO:0005200	Structural constituent of cytoskeleton	4/39	112/18496	8.90E-05	4.54E-03	2.59E-03
MF	GO:0005539	Glycosaminoglycan binding	5/39	240/18496	1.42E-04	5.42E-03	3.10E-03
MF	GO:0002020	Protease binding	4/39	142/18496	2.22E-04	6.80E-03	3.88E-03
KEGG	hsa05205	Proteoglycans in cancer	7/31	204/8546	6.44E-06	3.16E-04	2.54E-04
KEGG	hsa04512	ECM-receptor interaction	5/31	89/8546	1.50E-05	4.90E-04	3.95E-04
KEGG	hsa00600	Sphingolipid metabolism	4/31	54/8546	3.95E-05	9.67E-04	7.79E-04
KEGG	hsa04142	Lysosome	5/31	133/8546	1.04E-04	2.04E-03	1.64E-03

GO and KEGG enrichment analyses were performed to explore the biological roles of the 39 GRDEGs in DR, focusing on BP, CC, MF, and associated KEGG pathways. The GO analysis indicates that these 39 GRDEGs are mainly associated with numerous biological processes. GO, gene ontology; BP, biological process; CC, cellular component; MF, molecular function; KEGG, kyoto encyclopedia of genes and genomes; GRDEGs, glycosylation-related differentially expressed genes.

A network diagram integrating BP, CC, MF, and KEGG categories was also developed based on the enrichment results (Figures 4B–E). In the diagram, the connections represent specific molecular associations, with larger nodes indicating a higher number of genes involved in the corresponding terms.

#### Establishment of a molecular recognition model for diabetic retinopathy

**FIGURE 5:**
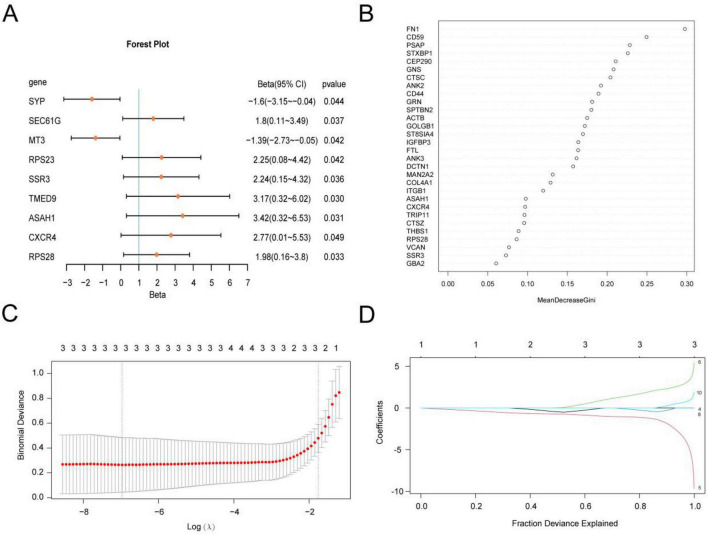
Molecular recognition model of DR. **(A)** Forest Plot of 9 GRDEGs included in the Logistic regression model in the molecular recognition model of DR. **(B)** MeanDecreaseGini scatter plot in the RF algorithm. **(C,D)** molecular recognition model plot **(C)** and variable trajectory plot **(D)** of LASSO regression model. DR, diabetic retinopathy; GRDEGs, glycosylation-related differentially expressed genes; LASSO, least absolute shrinkage and selection operator.

To assess the diagnostic potential of the 39 GRDEGs in DR, first, a logistic regression model was constructed using these 39 GRDEGs and visualized using a forest plot (Figure 5A). The model identified nine GRDEGs as significant predictors (*p* <0.05).

Next, an RF analysis was performed based on the 39 GRDEGs. In the RF algorithm, the top 10 genes were ranked by descending MeanDecreaseGini values as follows: *FN1*, *CD59*, *PSAP*, *STXBP1*, *CEP290*, *GNS*, *CTSC*, *ANK2*, *CD44*, and *GRN.* These genes were visualized using a MeanDecreaseGini scatter plot (Figure 5B).

Subsequently, LASSO regression was applied to establish the final molecular recognition model for DR using the 10 GRDEGs identified through RF analysis. For visualization, a diagram of the LASSO regression model was constructed (Figure 5C), along with a trajectory plot of the LASSO variables (Figure 5D). The LASSO regression model ultimately incorporated three GRDEGs—*CEP290*, *GNS*, and *GRN*—as model genes for predicting DR.

#### Internal validation and friends analysis of the molecular recognition model for diabetic retinopathy

**FIGURE 6:**
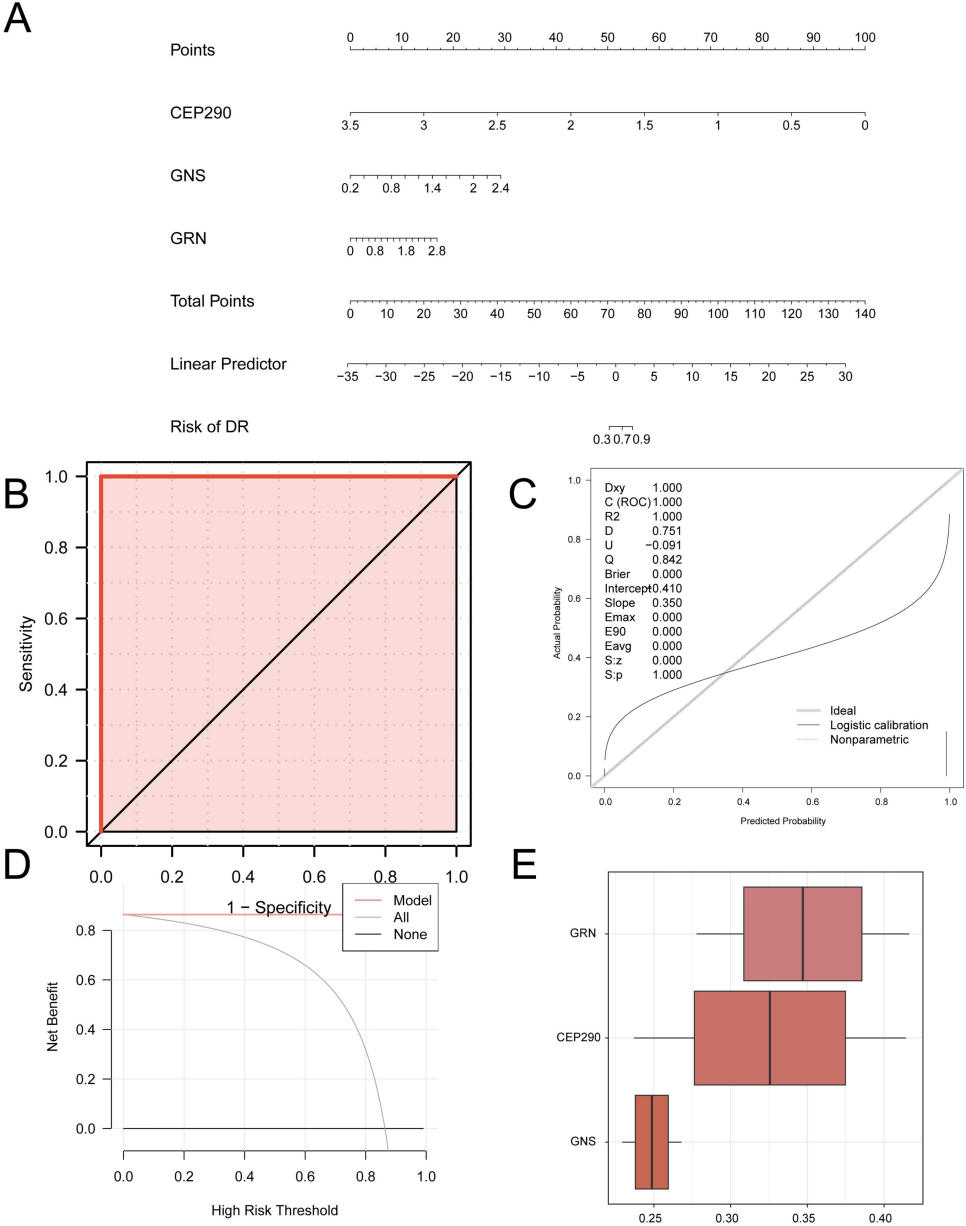
Diagnostic and validation analysis of DR and friends analysis. **(A)** Nomogram of Model Genes in GEO dataset in DR molecular recognition model. **(B)** GSE102485 GEO dataset of ROC curve. **(C,D)** Calibration Curve plot **(C)** and DCA plot **(D)** of Model Genes in GEO dataset for the diagnosis of DR. **(E)** Box plot of functional similarity (Friends) analysis results of Model Genes. The ordinate of the DCA plot is the net benefit, and the abscissa is the Probability Threshold or Threshold Probability. DR, diabetic retinopathy; DCA, decision curve analysis; ROC, receiver operating characteristic; AUC, area under the Curve. AUC is extremely accurate in the range of 0.9–1.

To further validate the molecular recognition model for DR, a nomogram was constructed using the model genes to illustrate their individual contributions to the GSE102485 dataset (Figure 6A). The results indicated that *CEP290* exhibited superior diagnostic performance compared to the other model genes, whereas *GRN* contributed the lowest. An ROC curve was generated using the R package pROC (Version 1.18.5) based on predicted probabilities derived from the logistic regression analysis of the GSE102485 dataset. The ROC curve (Figure 6B) showed that the logistic regression model achieved high classification accuracy for DR samples, with an AUC exceeding 0.9.

To assess the reliability of the molecular recognition model, a calibration curve was generated through calibration analysis. The curve shows comparisons between the predicted probabilities and actual outcomes (Figure 6C). Although the calibration line (dotted) deviated slightly from the ideal diagonal line, it remained relatively close, indicating that the model calibration was acceptable. Additionally, A DCA (differential expression analysis) experiment was conducted on the model genes, providing preliminary clues for the subsequent exploration of their potential value in real-world diagnosis and treatment processes (Figure 6D). The results showed that, within a specific threshold range, the decision curve of the model provided a greater net benefit than either the “All positive” or “All negative” strategies, highlighting the potential clinical applicability of the model.

Finally, the functional similarity (friends) score was used to identify genes with key roles in BPs associated with DR (Figure 6E). The results indicate that *GRN* plays a significant role in the context of DR. The model constructed using GSE102485 demonstrated favorable discriminatory performance in the training set, with the ROC curve indicating certain capacity to distinguish DR from control samples. Given the limited sample size and imbalanced group distribution in the training set, this result primarily reflects the model’s performance under the current data structure, warranting cautious interpretation.

#### External validation of the molecular recognition model for diabetic retinopathy

**FIGURE 7:**
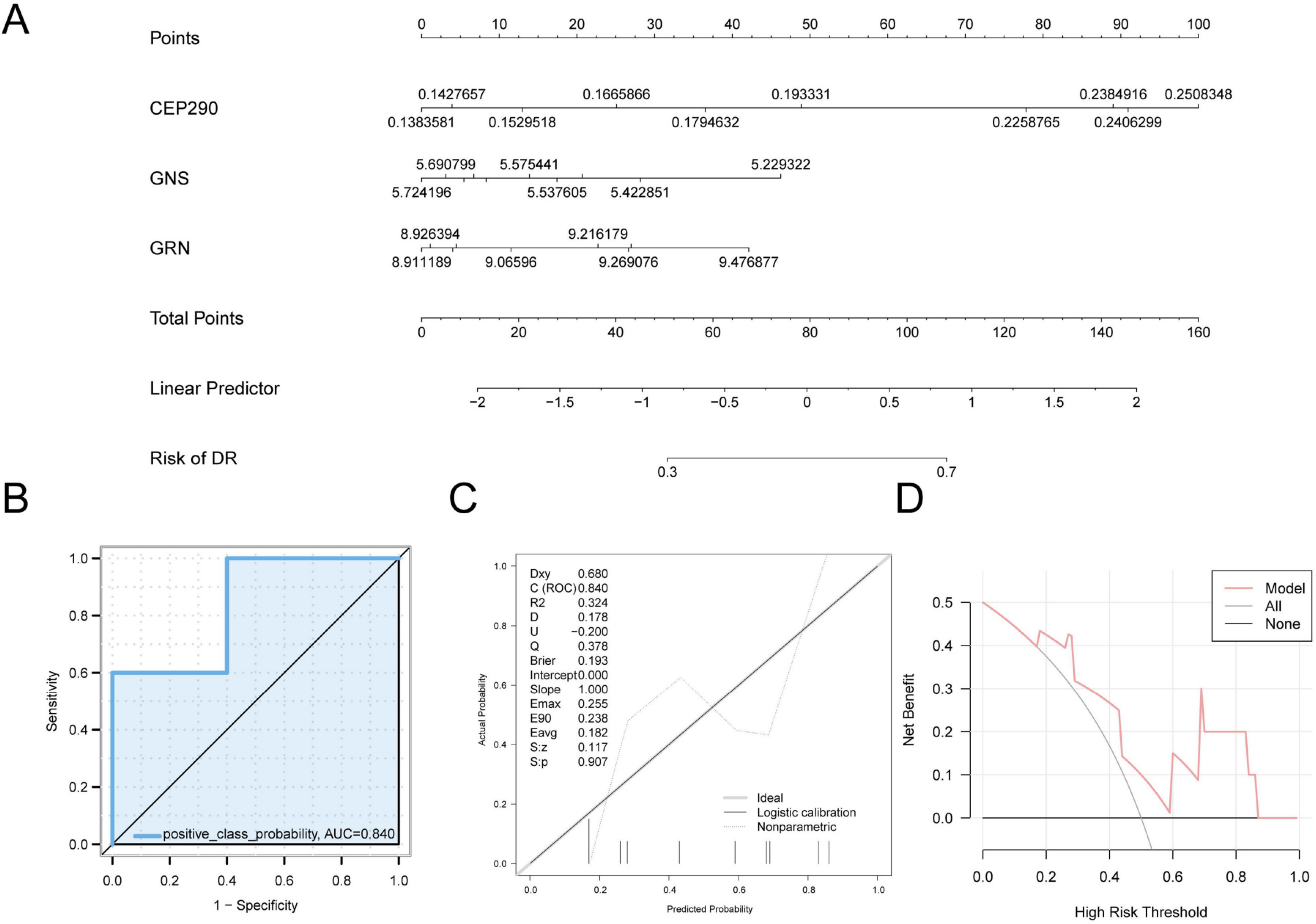
Diagnostic and validation analysis of DR. **(A)** Nomogram of Model Genes in GEO dataset in DR molecular recognition model. **(B)** ROC curve in GEO dataset GSE185011. **(C,D)** Calibration Curve plot **(C)** and DCA plot **(D)** of Model Genes in GEO dataset for the diagnosis of DR. The ordinate of DCA plot is the net benefit, and the abscissa is the Probability Threshold or Threshold Probability. DR, diabetic retinopathy; DCA, decision curve analysis; ROC, receiver operating characteristic; AUC, area under the curve. AUC has some accuracy in the range of 0.7–0.9.

To further evaluate the diagnostic performance of the DR model, a nomogram was constructed using the model genes to illustrate their interrelationships within the GEO dataset GSE185011 (Figure 7A). The results indicated that the diagnostic efficacy of *CEP290* surpassed that of other variables. Conversely, the expression level of *GRN* in the context of DR diagnosis was markedly lower than the contributions of other variables. The ROC curve was generated using the R package pROC (Version 1.18.5) based on predicted probabilities derived from the logistic regression analysis of the GSE185011 dataset. As shown in Figure 7B, the ROC curve showed that the logistic regression model exhibited moderate accuracy in distinguishing DR samples from control samples, with an AUC ranging from 0.7 to 0.9.

To assess the accuracy and discriminative ability of the DR molecular recognition model, a calibration curve was developed through calibration analysis. The predictive performance of the model was evaluated by examining the concordance between the predicted and actual probabilities under various conditions (Figure 7C). The calibration curve revealed that the calibration line of the model (dotted) deviated slightly from the ideal diagonal but remained relatively close. Furthermore, DCA was performed to evaluate the contribution of the model genes to the clinical applicability of the DR molecular recognition model (Figure 7D). The DCA curve showed that within a specific threshold range, the model provided greater net benefit than both the “All positive” and “All negative” strategies, thereby supporting its practical value and clinical utility. In the independent dataset GSE185011, the model retained moderate discriminatory performance, suggesting partial consistency of the model genes across different sample types. Given that the training set was derived from retinal tissue while the external validation set was from peripheral blood, this finding should be interpreted as exploratory cross-tissue evidence rather than confirmation of robust generalizability. The stability and clinical applicability of these results require further evaluation in larger-scale, independent populations with balanced sample distributions.

#### Verification of differential expression and ROC curve analysis for model genes in the training dataset GSE102485

**FIGURE 8:**
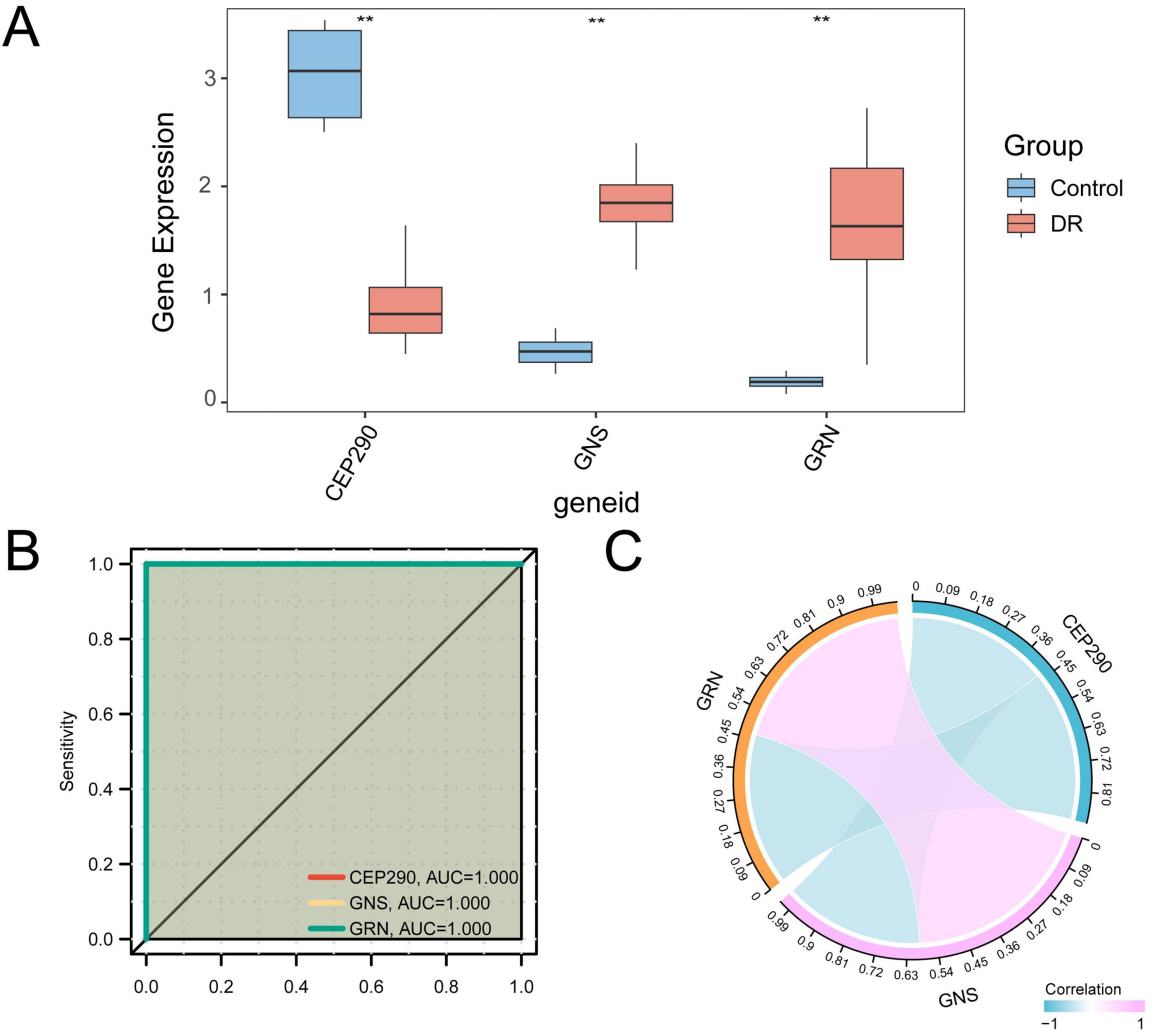
Differential expression validation and ROC curve analysis. **(A)** Group comparison diagram of Model Genes in DR group and Control group samples of GEO dataset GSE102485. **(B)** ROC curves of Model Genes, CEP290, GNS, GRN in the training set GSE102485. **(C)** Correlation and chord diagram of three Model Genes in dataset GSE102485. ** Represents a *p* <0.01, which is highly statistically significant. The absolute value of correlation coefficient (*r*-value) below 0.3 was weak or no correlation, between 0.3 and 0.5 was weak correlation, between 0.5 and 0.8 was moderate correlation, and above 0.8 was strong correlation. Red represents positive correlation; blue represents negative correlation. When AUC >0.5, it indicates that the expression of the molecule is a tendency to promote the event, and the closer the AUC is to 1, the better the diagnostic effect. The AUC was extremely accurate in the range of 0.9–1. DR, diabetic retinopathy; ROC, receiver operating characteristic; AUC, Area Under the Curve. Blue represents Control samples and orange represents DR samples.

Figure 8A compares the expression levels of three model genes between DR samples and controls in the GSE102485 dataset to evaluate their differential expression. The results indicated that all three model genes showed significant differences in expression between the DR and control groups (*p* <0.01).

Subsequently, the R package pROC (Version 1.18.5) was used to generate ROC curves based on the expression levels of the significantly differentially expressed model genes. As shown in Figure 8B, the ROC analysis revealed that these three model genes exhibited high classification performance in distinguishing DR samples from control samples, with AUC values ranging from 0.9 to 1. Finally, the relationships among the three model genes in the GSE102485 dataset were assessed through correlation analysis, and the results were visualized using both a correlation matrix and a chord diagram (Figure 8C). The analysis revealed that the three model genes primarily exhibited negative correlations with one another. ROC curve analysis indicated that the model effectively distinguished DR from control samples in the discovery dataset. However, the relatively small number of control samples in the training set introduces a risk of optimistic bias in model performance evaluation. Therefore, these results are better interpreted as reflecting discrimination under the current dataset structure rather than robust generalizability. ROC analysis in the blood dataset showed that the model had moderate discriminatory ability, but given transcriptional differences between tissue origins, this mainly reflects consistency in expression trends rather than validation of shared biological mechanisms between tissues.

#### Immune infiltration analysis of diabetic retinopathy (CIBERSORT)

**FIGURE 9:**
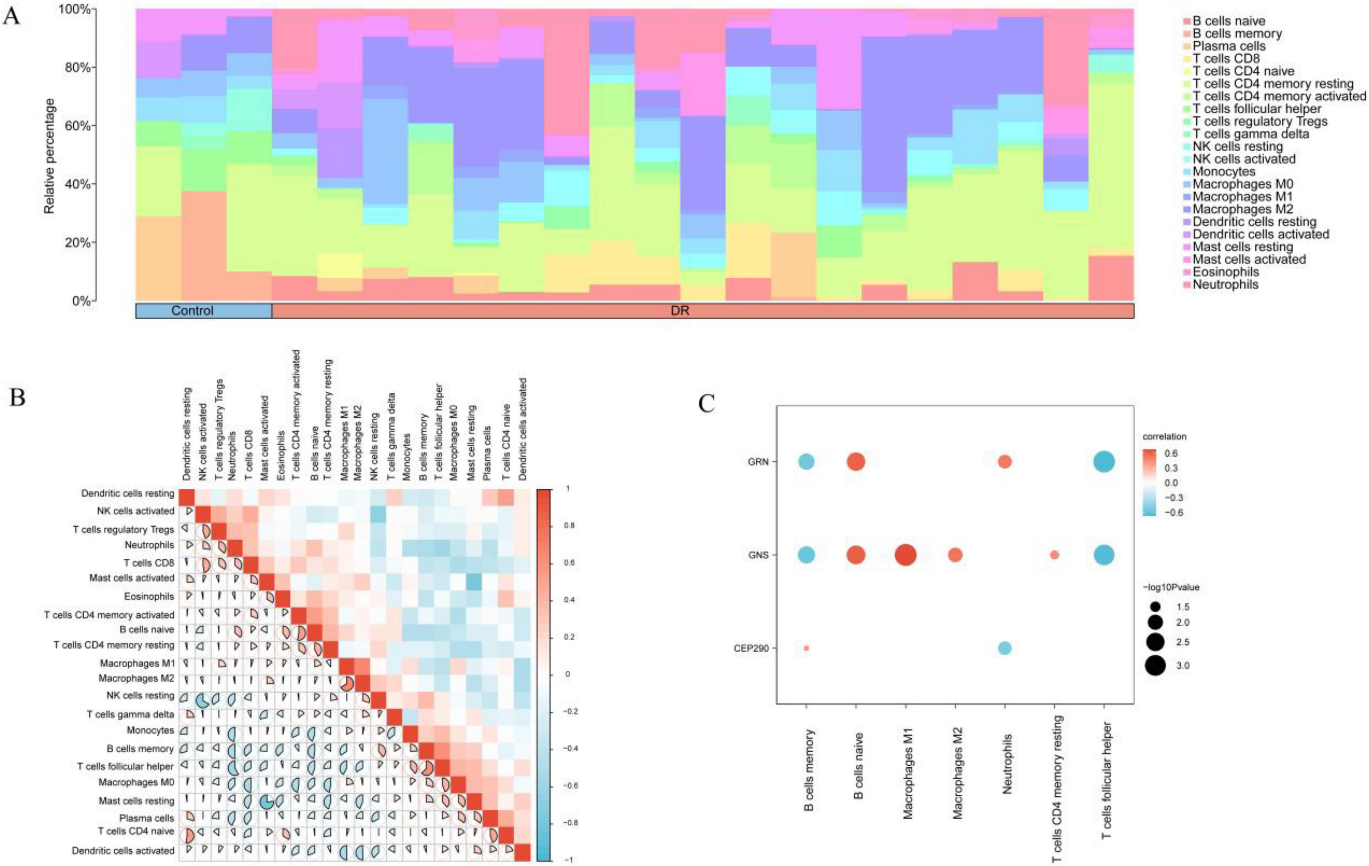
GSE102485 datasets immune infiltration analysis by CIBERSORT algorithm. **(A)** Bar chart of the proportion of immune cells in the GEO dataset. **(B)** Correlation heatmap of immune cell infiltration abundance in GEO dataset. **(C)** Bubble plot of correlation between Model Genes and immune cell infiltration abundance in GEO dataset. DR, Diabetic Retinopathy. The absolute value of correlation coefficient (*r*-value) below 0.3 was weak or no correlation, between 0.3 and 0.5 was weak correlation, between 0.5 and 0.8 was moderate correlation, and above 0.8 was strong correlation. Blue is negative correlation, red is positive correlation, and the depth of color represents the strength of the correlation. Blue represents Control samples and orange represents DR samples.

The CIBERSORT algorithm was used to estimate the relative proportions of 22 immune cell types in the GSE102485 dataset. Following the immune infiltration analysis, a bar chart was plotted to illustrate the distribution of these immune cells across the dataset (Figure 9A).

A correlation heat map was constructed to visualize the relationships among the 22 immune cell types (Figure 9B). The analysis revealed a strong negative correlation between resting mast cells and activated mast cells (*r* = −0.767, *p* <0.05). Conversely, M1 and M2 macrophages exhibited the strongest positive correlation, with a correlation coefficient of 0.665 (*p* <0.05). Additionally, a correlation bubble plot was created to examine the relationships between the model genes and the levels of immune cell infiltration in the dataset (Figure 9C). This plot showed a significant negative correlation between the model gene *GNS* and follicular helper T cells (*r* = −0.671, *p* <0.05), whereas model gene J was positively correlated with M1 macrophages (*r* = 0.676, *p* <0.05). However, the LM22 feature matrix used in this study was developed from signatures of 22 immune cell types derived from peripheral blood and is therefore primarily applicable to blood or blood-derived samples. Accordingly, its application to retinal tissue (GSE102485) was exploratory and aimed at characterizing relative trends in immune-related signals in DR, rather than quantitatively estimating actual immune cell proportions.

## Discussion

Diabetic retinopathy (DR) is a severe microvascular complication that significantly impairs vision and may lead to blindness, posing substantial burdens on individual health and public healthcare systems (25). Currently, diagnosis relies primarily on dilated fundus examination; however, the absence of early warning symptoms often results in delayed detection (26). This study investigates the role of GRDEGs in DR pathogenesis. By integrating multiple gene expression datasets, we identified 39 GRDEGs significantly associated with DR and developed a molecular recognition model. Retinal neovascular membrane samples served as the discovery dataset, with peripheral blood samples used for independent validation to preliminarily assess model generalizability across tissue sources. Notably, given the substantial transcriptional differences between retinal tissue and peripheral blood, these validation results reflect tissue transferability rather than hematological diagnostic potential.

The identification of 39 GRDEGs represents a significant advancement in understanding DR and highlights several potential therapeutic targets. Notable candidate genes include *RPS23, VCAN, ST8SIA4*, and *TMED9* on chromosome 5, as well as *CTSC, SPTBN2, CD44*, and *CD59* on chromosome 11. These genes warrant further investigation, particularly considering their involvement in other ocular diseases, which may reveal shared mechanisms or unique pathophysiological processes in DR. *RPS23* (ribosomal protein S23) is a direct target gene of miR-542-3p and is involved in regulating the stability of p53 (27). In DR, abnormal expression of p53 is closely associated with cell apoptosis and angiogenesis. *RPS23* may influence retinal cell survival and vascular integrity through its regulation of p53 (28). In other ocular diseases, such as retinal detachment and retinal vascular occlusion, abnormal expression of *RPS23* may exacerbate cellular damage, indicating its broad role in ocular pathology (28, 29). *VCAN* (versican) (30) is an aggregating chondroitin sulfate proteoglycan involved in cell adhesion, proliferation, migration, and angiogenesis. In DR, the aberrant expression of *VCAN* may contribute to retinal neovascularization and subsequent vision loss. *VCAN* interacts with components of the extracellular matrix (ECM), such as *TSG-6* and fibronectin, thereby influencing the retinal microenvironment (31). *ST8SIA4* (ST8α-N-acetyl-neuraminic acid synthase 4) has been implicated in immune cell infiltration and modulation of the tumor microenvironment (32). In DR, it may influence retinal inflammation and angiogenesis through its effects on cell adhesion and immune responses (33, 34). *TMED9* (transmembrane emp24 domain-containing protein 9) (35) is involved in regulating viral infections and immune responses. In DR, it may modulate retinal immune responses, potentially linking immune dysregulation and ocular infections to disease progression (36). *CTSC* (cathepsin C) (37) is a lysosomal protease implicated in cellular degradation and autophagy. Its dysregulation in DR may influence cell autophagy and apoptosis, ultimately affecting retinal cell survival (38). *SPTBN2* (spectrin beta, non-erythrocytic 2) (39, 40) has been associated with the regulation of angiogenesis (41). *CD44* is a cell surface molecule involved in cell adhesion and migration (42). Its upregulation can facilitate endothelial cell adhesion and migration, promoting pathological neovascularization in DR (43). *CD59* is a complement regulatory protein that modulates immune responses (44). Dysregulation of *CD59* may contribute to aberrant complement activation and inflammation in DR (45). Collectively, these genes are involved in key processes, such as proliferation, migration, adhesion, immunity, and inflammation, all of which are central to DR pathogenesis. Therefore, they represent potential targets for therapeutic interventions.

Our research indicates that GRDEGs are significantly enriched in BPs such as wound healing and sphingolipid metabolism, as well as in pathways including proteoglycans in cancer and lysosome-related signaling. Wound-healing-related BPs are in DR because chronic low-grade inflammation and repeated vascular injury shift the retina into a “persistent repair” mode, where excessive extracellular matrix deposition and fibroblast-to-myofibroblast conversion produce fibrovascular membranes that characterize proliferative DR. Sphingolipid metabolism is co-activated; ceramide-enriched membranes destabilize endothelial tight junctions and amplify VEGF signaling, thereby coupling abnormal wound repair with angiogenic sprouting. Proteoglycans-in-cancer signatures mirror the DR microenvironment: aberrant heparan-sulfate and versican networks sequester pro-angiogenic growth factors, promote macrophage M2 polarization and provide a scaffolding for invading endothelial cells, accelerating neovascular tuft formation (46). Lysosomal pathways are hyperactive in DR retinal pigment epithelium and macrophages, reflecting both increased clearance of oxidized photoreceptor debris and NLRP3 inflammasome-driven IL-1β release that sustains vascular leakage and retinal edema. Thus, each GRDEG-enriched pathway feeds a self-perpetuating loop of inflammation, abnormal repair and angiogenesis that underlies DR progression (46, 47). Under normal conditions, the wound healing process in the retina is usually static, given the low incidence of retinal injury (47). However, DR induces retinal microvascular damage and neurodegenerative changes, triggering responses similar to those of wound healing, including inflammatory cell infiltration, ECM remodeling, and neovascularization (47, 48). These processes are often abnormal in DR and may lead to retinal fibrosis and the development of proliferative lesions. In normal retinal tissues, sphingolipids are involved in cell signaling, membrane stability, and intercellular interactions. Although sphingolipid metabolism is typically stable, it becomes disrupted under hyperglycemic conditions, leading to metabolite accumulation and cellular dysfunction. Specific sphingolipid metabolites may increase oxidative stress and inflammation, thereby accelerating DR-related retinal damage (49). Proteoglycans in normal retinal tissue contribute to ECM integrity and cell signaling, maintaining retinal structure and function (50). In DR, alterations in their expression and function may promote inflammation, neovascularization, and ECM remodeling, thereby further advancing lesion development and fibrosis (51). The changes in proteoglycans not only affect matrix remodeling, but also regulate the secretion of growth factors and MMPs, promote the development of new blood vessels, and play a crucial role in the vitreous fibrosis of proliferative diabetic retinopathy (51). Lysosomes are crucial for cellular homeostasis, mediating processes such as signal transduction, metabolism, proliferation, differentiation, secretion, and protein/organelle quality control (52). Their involvement in DR pathogenesis suggests a role in the complex physiological processes underlying the disease (53). Additional metabolic pathways are also implicated in the pathogenesis of DR. Inflammation plays a key role, with interleukin (IL)-17A activating the JAK/STAT pathway and contributing to retinal damage (54). Overexpression of miR-224-5p has been shown to suppress the expression of IL-1β, IL-6, and TNF-α in high-glucose-induced human retinal pigment epithelial (ARPE-19) cells, thereby inhibiting the inflammatory response through the IL6ST/JAK/STAT pathway (55). Additionally, the JAK inhibitor AG490 has been shown to mitigate early diabetic retinal damage by reducing the expression of phosphorylated JAK2 and STAT3, thereby effectively inhibiting the JAK/STAT pathway (56).

This study identified 22 distinct immune cell infiltration patterns through CIBERSORT analysis. Notably, a negative correlation was observed between resting mast cells and activated mast cells, whereas M1 macrophages showed a positive correlation with M2 macrophages. Resting mast cells are primarily involved in maintaining tissue homeostasis and defense, whereas activated mast cells contribute to inflammation and immune regulation. In inflammatory diseases, the number and activity of activated mast cells typically increase, whereas resting mast cells decrease. These changes in the immune microenvironment may influence DR progression, providing a potential basis for immunotherapy (57). Immune cells, including microglia, T cells, neutrophils, and macrophages, play complex roles in the repair of retinal injury (58). Microglia, the resident immune cells in the retina, are activated early in DR and release proinflammatory cytokines (IL-1β, TNF-α, and IL-6), which disrupt the blood–retinal barrier and promote macrophage infiltration (59). Macrophages further exacerbate inflammation by releasing inflammatory mediators and may induce retinal neuronal apoptosis through interactions with microglia (60). T cells also contribute significantly to DR pathogenesis by infiltrating the retina and interacting with microglia, thereby intensifying inflammation. Regulatory T cells are essential for retinal repair, and their deficiency can hinder this process. T-cell infiltration can also activate microphages, creating a vicious cycle. In proliferative DR, the infiltration of macrophages and microglia is closely associated with neovascularization (61). Macrophages promote angiogenesis by secreting growth factors such as VEGF, which can further damage the retinal structure. Interactions among microglia, macrophages, and T cells form a complex inflammatory network that drives DR progression, contributing to both angiogenesis and neurodegeneration. Anti-inflammatory therapies may help regulate blood glucose levels, mitigate microvascular complications, and improve insulin resistance (62). Notably, immune infiltration analysis in this study was based on the LM22 reference matrix from CIBERSORT, which was originally derived from peripheral blood immune cells and has limited applicability to retinal tissue. Therefore, immune infiltration results from retinal samples reflect relative changes in immune-related molecular signals rather than quantitative estimates of true immune cell distributions. Although the findings indicate a trend toward immune cell activation and enhanced inflammatory responses in DR, further validation is warranted using retinal-specific immune reference matrixes, single-cell sequencing, or spatial transcriptomics. Therefore, the results should be interpreted in the context of these methodological limitations.

In this study, we developed a LASSO regression-based molecular recognition model for DR, integrating three differentially expressed glycosylation-related genes (GRDEGs): CEP290, GNS, and GRN. The model demonstrated favorable discriminatory performance in the training dataset, with AUC values exceeding 0.9, and retained moderate discriminatory ability in an independent blood dataset. Among these genes, GRN is particularly notable for its potential involvement in DR pathogenesis. The granulin protein encoded by GRN is a secreted factor implicated in cell proliferation, inflammatory responses, and tissue repair (63). Given that inflammation plays a central role in DR pathogenesis, GRN may influence disease progression by regulating inflammatory cell activity and proinflammatory mediator secretion. Additionally, GRN may promote angiogenesis in DR through interactions with vascularization-related pathways, such as the VEGF signaling pathway (63, 64), and may affect retinal cell proliferation and migration—critical processes in DR progression. CEP290, GNS, and GRN may participate in glycosylation-related pathways during DR progression through multiple mechanisms. CEP290, a ciliary structural protein, plays a regulatory role in ciliary signal transduction and photoreceptor structural integrity, with glycosylation modifications potentially essential for these functions. GNS catalyzes lysosomal glycosaminoglycan degradation and maintains glycosylation metabolic homeostasis; its dysfunction may lead to glycosaminoglycan accumulation and abnormal extracellular matrix remodeling. GRN is a secreted glycoprotein whose glycosylation status may affect protein stability and secretion, thereby modulating inflammatory responses and angiogenesis. Collectively, these proteins may form an interaction network linking glycosylation-dependent signaling regulation, metabolic homeostasis, and immune-inflammatory responses, potentially contributing to DR onset and progression (64).

Current DR diagnostic approaches primarily rely on fundoscopic examinations and imaging techniques, which often fail to detect early pathological changes, potentially resulting in delayed diagnosis. The gene-based molecular recognition model presented here may offer complementary sensitivity and specificity, potentially enabling earlier detection. However, these findings should be viewed as early proof-of-concept that the signature can distinguish disease spectra, not as definitive evidence of clinical utility. The limited sample size in the discovery cohort and the imbalance between case and control groups may affect model parameter stability and increase overfitting risk. Although external datasets were used for preliminary assessment, the tissue source discrepancy between training (retinal) and validation (blood) samples suggests these results should be interpreted as exploratory evidence of partial cross-tissue gene expression consistency, rather than confirmation of biological mechanisms or clinical applicability. Model robustness and clinical utility require prospective validation in larger, multicenter, and more balanced populations before any patient-level decision support can be contemplated.

This study has several limitations: small, retrospective GEO cohorts (65); lack of functional or longitudinal validation (66); and potential batch effects (67). Multicenter data, wet-lab assays, and prospective follow-up are essential before clinical application (68, 69). External validation in blood samples provides preliminary evidence of cross-tissue transferability. However, given substantial differences in cellular composition and transcriptional profiles between retinal tissue and peripheral blood, these results should be considered exploratory. They suggest that glycosylation-related gene signatures may retain partial discriminative signals across tissues, rather than demonstrating direct hematological diagnostic utility. Tissue specificity and clinical relevance must be confirmed using homologous tissues and prospective cohorts.

In summary, our identification of glycosylation-related differentially expressed genes (GRDEGs) advances understanding of DR molecular mechanisms, suggesting their potential involvement in DR pathogenesis. The molecular recognition model constructed based on CEP290, GNS, and GRN demonstrated favorable discriminatory performance in the training dataset and retained moderate discriminatory ability in an independent blood dataset. Despite ethical constraints and limited sample availability, the model exhibited certain discriminative capacity in the current data. Given the constraints in sample size and validation conditions, these findings should be interpreted as exploratory evidence primarily providing clues for subsequent mechanistic studies and independent population validation, rather than as confirmed therapeutic targets or diagnostic biomarkers. Future studies will expand sample sources, incorporate multicenter datasets, and evaluate early detection potential using clinically accessible samples (e.g., peripheral blood, aqueous humor) to better assess translational value.

## Data Availability

Publicly available datasets were analyzed in this study. This data can be found here: GEO repository (https://www. ncbi.nlm.nih.gov/geo/) include the following datasets: GSE102485 (https://www.ncbi.nlm.nih.gov/geo/query/acc.cgi?acc=GSE102485), GSE185011 (https://www.ncbi.nlm.nih.gov/geo/query/acc.cgi?acc=GSE185011). Further inquiries can be directed to the corresponding author.

## Footnotes

1
https://www.ncbi.nlm.nih.gov/geo/

2
https://www.genecards.org/

3
https://pubmed.ncbi.nlm.nih.gov/
