# Supplementary material for: Glycosylation gene-based molecular recognition model for diabetic retinopathy
Source: Front Med (Lausanne). 2026 May 18;13:1721094. doi: 10.3389/fmed.2026.1721094 (PMC13224470; doi:10.3389/fmed.2026.1721094)
Supplement: Supplementary file 1 [file Data_Sheet_1.docx]

rm(list=ls())

gc()

dir.create("output")

library(tidyverse)

library(randomForest)

## 数据输入

dat_expr <- read.csv("input/GSE102485_Datasets_Matrix.csv", row.names = 1, check.names = F)

dat_group <- read.csv("input/GSE102485_Datasets_Group.csv", check.names = F)

gene <- read.csv("input/5-GRDEGs.csv", check.names = F)

input <- dat_expr[gene$Gene, dat_group$ID] %>%

na.omit() %>% t() %>% as.data.frame()

input$group <- factor(dat_group$group, levels = c('Control', 'DR'))

## RF模型

set.seed(2024)

rf_res <- randomForest(

## 特征变量(除最后一列分组)

x = input[, -ncol(input)],

## 目标变量，group列，即想要预测的类别

y = input$group,

## 计算特征的重要性

importance = T,

## 决策树，通常更多的树可以提供更好的模型性能，但也会增加计算时间

ntree = 1000,

## 计算相似性矩阵

proximity = T

)

saveRDS(rf_res,file = "output/1-RF_res.rds")

## 提取RF结果的特征重要性

rf_importances0 <- importance(

## RF结果

rf_res,

## 不将度量划分为标准差

scale = F) %>%

## 转成数据框

as.data.frame()

## 根据MeanDecreaseGini筛选top10的基因

# MeanDecreaseAccuracy

rf_importances0 <- rf_importances0 %>%

arrange(desc(MeanDecreaseGini))

rf_importances <- head(rf_importances0,10)

RF_Genes <- rownames(rf_importances)

pdf(file = 'output/1-RF_MeanDecreaseGini.pdf', width = 8, height = 8)

varImpPlot(rf_res,

## 1表示准确率(Accuracy)，2表示节点纯度(Gini)

type = 2,

scale = F, main = '')

## 添加MeanDecreaseGini = 1的垂直线

abline(v = 0.21, col = "grey", lwd = 1, lty = 2)## 根据取top的最后一个值定义v

dev.off()

write.csv(RF_Genes, file = 'output/1-RF_Genes.csv',row.names = F)

# 1 环境变量准备----

## 1.1 清空环境----

rm(list=ls())

gc()

## 1.2 R包安装----

# 定义一个函数来检查并安装所需的R包

install_if_missing <- function(packages) {

installed_packages <- rownames(installed.packages())

for (pkg in packages) {

if (!(pkg %in% installed_packages)) {

BiocManager::install(pkg, dependencies = TRUE, update = FALSE)

}

}

}

# 检查安装的包列表

required_packages <- c("dplyr", "tibble",'forestplot')

# 调用函数进行检查和安装

install_if_missing(required_packages)

## 1.3 加载R包----

library(dplyr)

library(tibble)

library(forestplot)

## 1.4 创建文件夹----

dir.create("input/")

dir.create("output/")

# 2 读取输入文件----

# 读取表达矩阵

gene <- data.table::fread("input/5-GRDEGs.csv", data.table = F)

exp <- data.table::fread("input/GSE102485_Datasets_Matrix.csv", data.table = F)

gene <- as.data.frame(gene)

exp <- as.data.frame(exp)

# 提取RDEGs表达矩阵

exp <- exp[exp$V1 %in% gene$Gene,]

rownames(exp) <- exp$V1

exp <- exp[,-1]

sle <- as.data.frame(t(exp))

# 读取分组文件

group <- data.table::fread("input/GSE102485_Datasets_Group.csv", data.table = F)

colnames(group) <- c("geo_accession", "group")

table(group$group)

identical(rownames(sle),group$geo_accession)

# 3 单变量Logistic回归----

# 定义数据集处理进度函数

display.progress = function (index, totalN, breakN=20) {

if ( index %% ceiling(totalN/breakN) ==0 ) {

cat(paste(round(index*100/totalN), "% ", sep=""))

}

}

#一定保持对照组在前，疾病组在后才能使用下面这个代码，给对照组赋值0，疾病组赋值1

sle$Treat <- c(rep(0,sum(group$group == "Control")),

rep(1,sum(group$group == "DR")))

Logoutput <- c()

# 对每个RDEG进行逻辑回归并计算OR值和置信区间

# 森林图为OR值时运行（OR值没有负数，一定要注意）

for(i in 1:(ncol(sle)-1)){

# i = 1

display.progress(index = i,totalN = ncol(sle),breakN = 20)

g <- colnames(sle)[i]

mod1 <- glm(Treat~get(colnames(sle)[i]), family = binomial(link = 'logit'),data = sle)

fit <- summary(mod1)

se <- fit$coefficients[2,2]

beta <- as.numeric(fit$coefficients[,"Estimate"])[2]

Logoutput=rbind(Logoutput,data.frame(gene=g,

OR=beta %>% exp(),

OR_1=as.numeric(beta-1.96*se) %>% exp(),

OR_2=as.numeric(beta+1.96*se) %>% exp(),

pvalue=as.numeric(fit$coefficients[,"Pr(>|z|)"])[2],stringsAsFactors = F))

}

log.res <- Logoutput[which(Logoutput$pvalue < 0.05),"gene"]

Logoutput$`OR(95% CI)` <- paste0(round(Logoutput$OR,2),"(",round(Logoutput$OR_1,2),"~",round(Logoutput$OR_2,2),")")

range(Logoutput[,3:4])

fp <- Logoutput

fp <- fp[fp$gene %in% log.res,]

fp$pvalue <- round(fp$pvalue,6)

xr <- colnames(fp)

xr[2:4] <- NA

fp <- rbind(xr,fp)

fp[,2:4] <- apply(fp[,2:4],2,as.numeric)

p_fore <- forestplot(labeltext=as.matrix(fp[,c(1,6,5)]),

mean=fp$OR,

lower=fp$OR_1,

upper=fp$OR_2,

zero=1,

# 超出这个范围，端点用箭头表示

clip = c(0,round(max(fp$OR[-1])+as.numeric(quantile(fp$OR[-1], 0.25)))),

ci.vertices = T, # 森林图两个端点

boxsize=0.2,

lineheight = unit(7,'mm'),

colgap=unit(2,'mm'),

# 点型选择圆形

fn.ci_norm = fpDrawCircleCI,

col=fpColors(box='#F17F42',

summary='#8B008B',

lines = 'black',

zero = '#8EC0E4'),

xlab="OR",

lwd.zero=1.5,

lwd.ci=1.5,

lwd.xaxis = 1.5,

# lwd.yaxis = 2.5,

txt_gp = fpTxtGp(ticks = gpar(cex = 0.85),

xlab = gpar(cex = 0.8),

cex = 0.9),

lty.ci = "solid",

title = "Forest Plot",

line.margin = 0.08,

graph.pos=2)

dev.off()

pdf("output/1-Logistic_ForestPlot.pdf",height = 12.9,width = 8.1)

print(p_fore)

dev.off()

# # 如果OR值太大，改为展示Beta值（Beta值有正负）

for(i in 1:(ncol(sle)-1)){ # i = 1

display.progress(index = i,totalN = ncol(sle),breakN = 20)

g <- colnames(sle)[i]

mod1 <- glm(Treat~get(colnames(sle)[i]), family = binomial(link = 'logit'),data = sle)

fit <- summary(mod1)

se <- fit$coefficients[2,2]

beta <- as.numeric(fit$coefficients[,"Estimate"])[2]

Logoutput=rbind(Logoutput,data.frame(gene=g,

Beta=beta, #OR beta %>% exp()

Beta_1=beta-1.96*se, #as.numeric(beta-1.96*se)

Beta_2=beta+1.96*se, #as.numeric(beta+1.96*se)

pvalue=as.numeric(fit$coefficients[,"Pr(>|z|)"])[2],stringsAsFactors = F))

}

log.res <- Logoutput[which(Logoutput$pvalue < 0.05),"gene"]

Logoutput$`Beta(95% CI)` <- paste0(round(Logoutput$Beta,2),"(",round(Logoutput$Beta_1,2),"~",round(Logoutput$Beta_2,2),")")

range(Logoutput[,3:4])

fp <- Logoutput

fp <- fp[fp$gene %in% log.res,]

fp$pvalue <- round(fp$pvalue,6)

xr <- colnames(fp)

xr[2:4] <- NA

fp[, 5] <- ifelse(fp[, 5] >= 0.001,

formatC(fp[, 5], format = "f", digits = 3, drop0trailing = F),

"< 0.001")

fp <- rbind(xr,fp)

fp[,2:4] <- apply(fp[,2:4],2,as.numeric)

p_fore <- forestplot(labeltext=as.matrix(fp[,c(1,6,5)]),

mean=fp$Beta,

lower=fp$Beta_1,

upper=fp$Beta_2,

zero=1,

ci.vertices = T, #森林图两个端点

boxsize=0.2,

lineheight = unit(7,'mm'),

colgap=unit(2,'mm'),

fn.ci_norm = fpDrawCircleCI, #点型选择圆形

col=fpColors(box='#F17F42',

summary='#8B008B',

lines = 'black',

zero = '#8EC0E4'),

xlab="Beta", #"Beta" 如果OR值过大替换为beta

lwd.zero=2.5, # 设置 zero 线宽为 0.75

lwd.ci=2.5, #置信度线宽

lwd.xaxis = 2.5, # 设置x轴线宽

# lwd.yaxis = 2.5, # 设置y轴线宽

txt_gp = fpTxtGp(ticks = gpar(cex = 0.85),

xlab = gpar(cex = 0.8),

cex = 0.9),

lty.ci = "solid",

title = "Forest Plot",

line.margin = 0.08,

graph.pos=2)

dev.off()

pdf("output/1-Logistic_ForestPlot-Beta.pdf",height = 7.9,width = 7.1)

print(p_fore)

dev.off()

# 4 输出文件----

write.csv(fp,"output/1-Logistic_Univarate_result.csv",row.names = F)

write.csv(log.res,"output/1-Logistic_Genes.csv",row.names = F)

# Copyright (C) 2011 John Colby

# http://github.com/johncolby/SVM-RFE

svmRFE.wrap <- function(test.fold, X, ...) {

# Wrapper to run svmRFE function while omitting a given test fold

train.data = X[-test.fold, ]

test.data = X[test.fold, ]

# Rank the features

features.ranked = svmRFE(train.data, ...)

return(list(feature.ids=features.ranked, train.data.ids=row.names(train.data), test.data.ids=row.names(test.data)))

}

svmRFE <- function(X, k=1, halve.above=5000) {

# Feature selection with Multiple SVM Recursive Feature Elimination (RFE) algorithm

n = ncol(X) - 1

# Scale data up front so it doesn't have to be redone each pass

cat('Scaling data...')

X[, -1] = scale(X[, -1])

cat('Done!\n')

flush.console()

pb = txtProgressBar(1, n, 1, style=3)

i.surviving = 1:n

i.ranked = n

ranked.list = vector(length=n)

# Recurse through all the features

while(length(i.surviving) > 0) {

if(k > 1) {

# Subsample to obtain multiple weights vectors (i.e. mSVM-RFE)

folds = rep(1:k, len=nrow(X))[sample(nrow(X))]

folds = lapply(1:k, function(x) which(folds == x))

# Obtain weights for each training set

w = lapply(folds, getWeights, X[, c(1, 1+i.surviving)])

w = do.call(rbind, w)

# Normalize each weights vector

w = t(apply(w, 1, function(x) x / sqrt(sum(x^2))))

# Compute ranking criteria

v = w * w

vbar = apply(v, 2, mean)

vsd = apply(v, 2, sd)

c = vbar / vsd

} else {

# Only do 1 pass (i.e. regular SVM-RFE)

w = getWeights(NULL, X[, c(1, 1+i.surviving)])

c = w * w

}

# Rank the features

ranking = sort(c, index.return=T)$ix

if(length(i.surviving) == 1) {

ranking = 1

}

if(length(i.surviving) > halve.above) {

# Cut features in half until less than halve.above

nfeat = length(i.surviving)

ncut = round(nfeat / 2)

n = nfeat - ncut

cat('Features halved from', nfeat, 'to', n, '\n')

flush.console()

pb = txtProgressBar(1, n, 1, style=3)

} else ncut = 1

# Update feature list

ranked.list[i.ranked:(i.ranked-ncut+1)] = i.surviving[ranking[1:ncut]]

i.ranked = i.ranked - ncut

i.surviving = i.surviving[-ranking[1:ncut]]

setTxtProgressBar(pb, n-length(i.surviving))

flush.console()

}

close(pb)

return (ranked.list)

}

getWeights <- function(test.fold, X) {

# Fit a linear SVM model and obtain feature weights

train.data = X

if(!is.null(test.fold)) train.data = X[-test.fold, ]

svmModel = svm(train.data[, -1], train.data[, 1], cost=10, cachesize=500,

scale=F, type="C-classification", kernel="linear")

t(svmModel$coefs) %*% svmModel$SV

}

WriteFeatures <- function(results, input, save=T, file='features_ranked.txt') {

# Compile feature rankings across multiple folds

featureID = sort(apply(sapply(results, function(x) sort(x$feature, index.return=T)$ix), 1, mean), index=T)$ix

avg.rank = sort(apply(sapply(results, function(x) sort(x$feature, index.return=T)$ix), 1, mean), index=T)$x

feature.name = colnames(input[, -1])[featureID]

features.ranked = data.frame(FeatureName=feature.name, FeatureID=featureID, AvgRank=avg.rank)

if(save==T) {

write.table(features.ranked, file=file, quote=F, row.names=F)

} else {

features.ranked

}

}

FeatSweep.wrap <- function(i, results, input) {

# Wrapper to estimate generalization error across all hold-out folds, for a given number of top features

svm.list = lapply(results, function(x) tune(svm,

train.x = input[x$train.data.ids, 1+x$feature.ids[1:i]],

train.y = input[x$train.data.ids, 1],

validation.x = input[x$test.data.ids, 1+x$feature.ids[1:i]],

validation.y = input[x$test.data.ids, 1],

# Optimize SVM hyperparamters

ranges = tune(svm,

train.x = input[x$train.data.ids, 1+x$feature.ids[1:i]],

train.y = input[x$train.data.ids, 1],

ranges = list(gamma=2^(-12:0), cost=2^(-6:6)))$best.par,

tunecontrol = tune.control(sampling='fix'))$perf)

error = mean(sapply(svm.list, function(x) x$error))

return(list(svm.list=svm.list, error=error))

}

PlotErrors <- function(errors, errors2=NULL, no.info=0.5,

ylim=range(c(errors, errors2), na.rm=T),

xlab='Number of Features', ylab='5 x CV Error') {

# Makes a plot of average generalization error vs. number of top features

AddLine <- function(x, col='#A7C1E7') {

lines(which(!is.na(errors)), na.omit(x), col=col,lwd=3)

points(which.min(x), min(x, na.rm=T), col='firebrick3')

text(which.min(x), min(x, na.rm=T), paste(which.min(x), '-',

format(min(x, na.rm=T), dig=3)), pos=2, col='red', cex=1.15)

}

plot(errors, type='n', ylim=ylim, xlab=xlab, ylab=ylab)

AddLine(errors)

if(!is.null(errors2)) AddLine(errors2, 'gray30')

abline(h=no.info, lty=2)

}

Plotaccuracy <- function(errors, errors2=NULL, no.info=0.5,

ylim=range(c(errors, errors2), na.rm=T),

xlab='Number of Features', ylab='5 x CV Accuracy') {

# Makes a plot of average generalization error vs. number of top features

AddLine <- function(x, col='#E8B9B1') {

lines(which(!is.na(errors)), na.omit(x), col=col,lwd=3)

points(which.max(x), max(x, na.rm=T), col='firebrick3')

text(which.max(x), max(x, na.rm=T), paste(which.max(x), '-',

format(max(x, na.rm=T), dig=3)), pos=2, col='red', cex=1.15)

}

plot(errors, type='n', ylim=ylim, xlab=xlab, ylab=ylab)

AddLine(errors)

if(!is.null(errors2)) AddLine(errors2, 'gray30')

abline(h=no.info, lty=2)

}

#LASSO

lasso_exp <- dat_expr[RF_Genes,dat_group$ID]

x <- as.matrix(t(lasso_exp))

y <- dat_group$group

library(glmnet)

set.seed(2024)

fit_lasso = glmnet(x, y, family = "binomial",alpha = 1,lambda = NULL)

pdf(file = "output/3-LASSO_Lambda.pdf",height = 5,width = 8.1)

plot(fit_lasso, xvar = "dev", label = TRUE)

dev.off()

cvfit = cv.glmnet(x, y, family = "binomial",nfolds = 5)

pdf(file = "output/2-LASSO_Likelihood.pdf",height = 5,width = 8.1)

plot(cvfit)

dev.off()

cvfit$lambda.min

cvfit$lambda.1se

myCoefs <- coef(cvfit, s="lambda.min")

lasso_fea<-myCoefs@Dimnames[[1]][which(myCoefs != 0 )]

lasso_fea<-lasso_fea[-1]

lasso_fea

write.csv(lasso_fea,"output/2-LASSO_Genes.csv",row.names = F)

#Risk Score计算和分组

library(dplyr)

tmp <- as.data.frame(t(lasso_exp[lasso_fea,]))#lasso基因的表达谱

lasso_coef <- myCoefs@x

lasso_coef <- cbind(lasso_fea,lasso_coef[-1])

lasso_coef <- as.data.frame(lasso_coef)

rownames(lasso_coef) <- lasso_coef$lasso_fea

lasso_coef$V2 <- as.numeric(lasso_coef$V2)#lasso模型系数

write.csv(lasso_coef,"output/3-LASSO_Coef.csv",row.names = F,quote = F)#输出系数

risk.score <- apply(tmp,1,function(x) {crossprod(as.numeric(x),lasso_coef[colnames(tmp),]$V2)})# 表达加权系数求和计算riskscore

tmp$RiskScore = risk.score

tmp1 <- tmp[dat_group[(dat_group$group == "DR"),]$ID,]

tmp1 <- tmp1 %>%

dplyr::mutate(risklevel = case_when(

RiskScore >= median(tmp1$RiskScore) ~ "HighRisk",

RiskScore < median(tmp1$RiskScore) ~ "LowRisk"

))

write.csv(tmp1,"output/4-LASSO_RiskGroup.csv",row.names = T,quote = F)

all(rownames(tmp) == dat_group$ID)

tmp$group = dat_group$group

write.csv(tmp,"output/4-LASSO_RiskScore_all.csv",row.names = T,quote = F)

## 计算验证集风险得分

GSE185011 <- data.table::fread("input/GSE185011_Datasets_Matrix.csv") %>% tibble::column_to_rownames("V1")

GSE185011_group <- data.table::fread("input/GSE185011_Datasets_Group.csv")

tmp2 <- data.frame(t(GSE185011[lasso_fea,GSE185011_group$ID]))

risk.score2 <- apply(tmp2,1,function(x) {crossprod(as.numeric(x),lasso_coef[colnames(tmp2),]$V2)})# 表达加权系数求和计算riskscore

tmp2$RiskScore = risk.score2

tmp2_1 <- tmp2[GSE185011_group[(GSE185011_group$Group == "DR"),]$ID,]

tmp2_1 <- tmp2_1 %>%

dplyr::mutate(risklevel = case_when(

RiskScore >= median(tmp2_1$RiskScore) ~ "HighRisk",

RiskScore < median(tmp2_1$RiskScore) ~ "LowRisk"

))

write.csv(tmp1,"output/5-GSE185011_RiskGroup.csv",row.names = T,quote = F)

all(rownames(tmp2) == GSE185011_group$ID)

tmp2$group = GSE185011_group$Group

write.csv(tmp2,"output/5-GSE185011_RiskScore_all.csv",row.names = T,quote = F)
